# Effects of 1-Methylcyclopropene and Modified Atmosphere Packaging on the Antioxidant Capacity in Pepper “Kulai” during Low-Temperature Storage

**DOI:** 10.1100/2012/474801

**Published:** 2012-07-31

**Authors:** Chung Keat Tan, Zainon Mohd Ali, Ismanizan Ismail, Zamri Zainal

**Affiliations:** ^1^School of Bioscience and Biotechnology, National University of Malaysia, Selangor, 43600 Bangi, Malaysia; ^2^Institute of System Biology (INBIOSIS), National University of Malaysia, Selangor, 43600 Bangi, Malaysia

## Abstract

The objective of the present study was to simultaneously evaluate the effect of a postharvest treatment on the pepper's antioxidant content and its ability to retain its economical value during the postharvest period. The fruits were pretreated by modified atmosphere packaging (MAP) with or without treatment with 1-methylcyclopropene (1-MCP) before cold storage at 10°C. Changes in the levels of non-enzymatic antioxidants, including the total phenolic, ascorbic acid levels and the total glutathione level, as well as enzymatic antioxidants, including ascorbate peroxidase (APX), glutathione reductase (GR), and catalase (CAT), were determined. Both treatments successfully extended the shelf life of the fruit for up to 25 days, and a high level of antioxidant capacity was maintained throughout the storage period. However, 1-MCP treatment maintained the high antioxidant capacity for a longer period of time. The 1-MCP-treated peppers maintained high levels of phenolic content, a high reduced glutathione (GSH)/oxidised glutathione (GSSG) ratio, decreased levels of ascorbic acid and CAT activity, and increased levels of APX and GR compared with the peppers that were not treated with 1-MCP. The overall results suggested that a combination of 1-MCP and MAP was the most effective treatment for extending shelf life while retaining the nutritional benefits.

## 1. Introduction

Peppers (*Capsicum annuum* “Kulai”) have been consumed for more than three centuries. In fact, most peppers have been used extensively as spices or condiments in Asian foods. The high market demand for peppers is due not only to their natural colours and spices but also to the nutritional benefits of the fruit, particularly because this fruit serves as an excellent source of dietary antioxidants [1]. The total antioxidant activities in peppers, which consist of enzymatic and non-enzymatic antioxidants, are in the highest range among the most commonly consumed vegetables [2]. The intake of foods with high levels of antioxidant constituents promotes health. Dietary antioxidants help protect against the harmful effects of reactive oxygen species (ROS) in our bodies and thus prevent human diseases, including cardiovascular disease and cancer, when adequate amounts are consumed daily [3]. However, the pepper is a perishable fruit with a short shelf life and, therefore, is highly susceptible to a rapid decrease in quality. Thus, peppers require a modified postharvest environment to reduce the nutritional loss.

Various antioxidants are present in peppers that act as suppressors against oxidative stress. Phenolic compounds, the main contributors to the antioxidant capacity of plants, have been studied often. Phenolic compounds are important for inhibiting lipid autoxidation by acting as radical scavengers [4], which, in turn, protect against the propagation of an oxidative chain. Peppers serve as excellent sources of vitamin C, with one pepper able to satisfy the daily requirement for this vitamin [5]. The high vitamin C content is important for chelating heavy metal ions, scavenging reactive radicals, and suppressing peroxidation, and these actions prevent the effects of degenerative diseases [6, 7]. Glutathione exists in a reduced form (GSH) and an oxidised form (GSSG). In both animals and plants, GSH is the predominant form that acts as an antioxidant and reducing buffer [8]. The enzymatic antioxidant system, which includes ascorbate peroxidase (APX, EC 1.11.1.11), catalase (CAT, EC 1.11.1.6), and glutathione reductase (GR, EC 1.6.4.2), is also synergistically involved with non-enzymatic antioxidants to reduce oxidative stress in plants.

The pepper “Kulai” is mainly harvested and consumed at the mature green stage or at the fully ripened/red stage. However, the market value of peppers is restricted by the postharvest environment due to the short shelf life of peppers [9]. Following the rise in demand from consumers for products of excellent quality and high nutritional value, optimisation of the postharvest conditions to retain the fruit quality is now a focus of current studies. Low-temperature treatment has often been used to prevent physical changes, and the suitable range for pepper storage is between 7°C and 13°C to avoid a chilling injury [10, 11]. Modified atmosphere packaging (MAP) with an optimal gas concentration was able to improve the quality retention of peppers with a low-temperature treatment and to control any postharvest disease [12]. An ethylene receptor blocker, 1-methylcyclopropene (1-MCP), has recently been utilised to extend the shelf life of some climacteric fruits, and a persistent effect was demonstrated [13]. The beneficial effects of 1-MCP include lower lipoxygenase activities and electrolyte leakage, prolonged cold storage life, delayed skin colour changes, and suppression of certain ethylene-induced postharvest physiological disorders [14–16].

Formal postharvest research that is available regarding the effects of a combination treatment (1-MCP and MAP) on the antioxidant capacity of the fruit is still limited, especially in peppers, because 1-MCP is still a new technology. The objective of the present study was to simultaneously evaluate the effect of a postharvest treatment on both the pepper's antioxidant content and the pepper's ability to retain its economical value during the postharvest period. It is anticipated that the results obtained from the current study will help clarify the possible interactions between and roles of the different treatments in regulating antioxidant activity. The findings should be important for developing an effective postharvest treatment to retain the nutritional benefits of the fruit. 

## 2. Materials and Methods

### 2.1. Materials

The pepper “Kulai” (*Capsicum annuum* cv. Kulai) was harvested at the Bukit Lanchong farm at the mature green and fully ripened/red stages. Fruits that were free from diseases were selected and washed with distilled water. All of the fruits were then air-dried and packed into 13 identical polyethylene bags (thickness: 50 *μ*m) such that each bag contained 100 uniform peppers. Three bags left unsealed served as controls, five sealed bags flushed with CO_2_/N_2_ mixture (25% CO_2_ + 75% N_2_) served as the MAP-treated group, and the remaining bags were treated with a combination of MAP (25% CO_2_ + 75% N_2_) and 90 ppb of gaseous 1-MCP (12 hr, 30°C) from SmartFresh (AgroFresh Inc., Spring House, PA) for 12 h [17]. Following the treatment, both the MAP-treated and MAP+1-MCP-treated groups were stored at 10°C for up to 25 days. The control group without MAP treatment (unsealed) or 1-MCP treatment was stored at room temperature and given the name UCRT (unsealed control at room temperature). After 21 days of storage, 2 bags from each MAP-treated and MAP+1-MCP-treated group were placed at room temperature for 5 days to simulate commercial shelf handling.

Fifty uniform peppers were sampled at specific intervals during the storage period (as indicated in the figures). The fruits were cut into small pieces, the seeds were discarded, and the fruit pieces were immediately frozen in liquid nitrogen. The tissues were stored at −80°C until further analysis. There were six replicates per time interval with 5 g of peppers per replicate. All of the enzymatic and non-enzymatic assays were performed based on these replicates.

Unless otherwise stated, all solvents, salts, and acids were purchased from Sigma Chemical Co. (St. Louis, USA). All of the reagents were of HPLC grade and of the highest purity available. All aqueous solutions were prepared with distilled water. 

### 2.2. Total Phenolic Content

The total phenolic content in the pepper fruits was measured using the method adapted from Singleton and Rossi [18]. Five grams of fresh weight of the pepper tissues was pulverised using a mortar and pestle with 20 mL of cold 100% methanol, and the samples were then centrifuged at 3,000 rpm and 4°C for 15 min. The extract was appropriately diluted and then oxidised with 100 *μ*L of freshly diluted 50% Folin-Ciocalteu reagent. After 3 min, this reaction was neutralised by adding 2 mL of 2% (w/v) sodium carbonate. After incubating for 30 min at room temperature, the absorbance of the resulting blue-coloured solution was determined at 750 nm using a UV-visible spectrophotometer (UV-160A, Shimadzu, Kyoto, Japan). A standard curve was prepared using the same procedure with gallic acid (10, 20, 50, and 100 mg/L). The total phenolic content of the pepper samples was expressed in milligrams of gallic acid equivalent (GAE) per gram of fresh weight (FW).

### 2.3. Ascorbic Acid Content

Five grams of the sample was extracted using 25 mL of HPLC-grade water. The homogenate was centrifuged at 13,000 rpm for 15 min. The supernatant was then filtered using a syringe filter (nylon membrane, 0.02 *μ*m). Quantification was achieved using an external standard method adapted from Lim et al. [19] with slight modification. HPLC analysis of ascorbic acid was performed using HPLC system equipped with a diode array detector (Prominence-20A, Shimadzu, Kyoto, Japan). The samples (20 *μ*L) were separated at 40°C on a Waters Symmetry C18 column (3.9 × 150 mm id; 5 *μ*m particle size) (Milford, MA, USA) using a mobile phase of 5% acetic acid at a flow rate of 1 mL/min. The amount of ascorbic acid was calculated from the absorbance at 254 nm using ascorbic acid (20, 40, 60, 80, and 100 mg/L) as a standard. The results are expressed as milligram of ascorbic acid per gram of FW. 

### 2.4. Total Glutathione Content

Five grams of the sample was homogenised in a cold mortar using 15 mL of 5% 5-sulphosalicylic acid. The homogenate was then centrifuged at 9,000 rpm for 15 min. The assay to determine the total glutathione content was based on the method of Anderson [20] with slight modification. The reaction mixture was composed of 700 *μ*L of 0.2 mM NADPH, 100 *μ*L of 6 mM DTNB, and 180 *μ*L of 0.143 M potassium phosphate buffer (pH 7.5). The mixture was incubated in a water bath at 30°C for 5 min before the addition of 20 *μ*L of the supernatant and 1.5 units of glutathione reductase. The change in absorbance at 412 nm during 1 min was monitored using a UV-visible spectrophotometer (UV-160A, Shimadzu, Kyoto, Japan). A standard curve was prepared using the same procedure with GSH equivalents (50, 100, 150, and 200 *μ*M). The total glutathione content of the pepper samples was expressed in *μ*mol per gram of FW.

For the GSSG determination, the supernatant was first diluted 10 times with 0.5 M potassium phosphate buffer (pH 6.5). Then, 20 *μ*L of 2-vinylpyridine was added to 1 mL of the mixture, which was followed by vigorous mixing. After one hour incubation at room temperature, 20 *μ*L of the mixture was removed and used for the glutathione assay described above. A standard curve was plotted using GSSG (25, 50, 75, and 100 *μ*M), and the results were expressed in *μ*mol per gram of FW. 

### 2.5. Enzyme Assays

The APX and CAT assays both used the same extraction procedure. For 5 g of sample tissue, 10 mL of phosphate buffer (0.1 M, pH 7.0), which consisted of 1.0 mM EDTA and 1% polyvinyl polypyrrolidone (PVP), was used as the extraction buffer. The method used for the analysis of the APX activity was adapted from Nakano and Asada [21]. The reaction mixture contained 1.91 mL of phosphate buffer (50 mM, pH 7.0), 0.05 mL of ascorbate (0.5 mM), 0.01 mL of hydrogen peroxide (H_2_O_2_) (0.1 mM), and 30 *μ*L of enzyme extract. The specific activity of APX was determined by monitoring the decline in the absorbance at 290 nm using a UV-visible spectrophotometer (UV-160A, Shimadzu, Kyoto, Japan) and was expressed as units per gram of fresh weight. One unit of APX was defined as the amount of enzyme that oxidised 1 *μ*mol of ascorbate per min at room temperature. 

The CAT activity was estimated according to the method of Beers and Sizer [22]. The reaction mixture consisted of 0.1 mL of enzyme extract, 50 mM phosphate buffer (pH 7.0), and 15 mM H_2_O_2_. The depletion of H_2_O_2_ was determined by measuring the change in the absorbance at 240 nm using a UV-visible spectrophotometer (UV-160A, Shimadzu, Kyoto, Japan). One unit of CAT was defined as the amount of enzyme needed to reduce 1 *μ*mol of H_2_O_2_ in 1 min. The specific activity was expressed as units per gram of FW.

GR was extracted from 5 g samples using 20 mL of cold potassium phosphate extraction buffer, which contained 1 mM EDTA, 0.5% Triton X-100, 0.1 mM 2-mercaptoethanol, and 2% PVP. The assay was adapted from the method of Bergmeyer [23] with a slight modification. The reaction mixture consisted of 0.2 M potassium phosphate buffer (pH 7.5), 2 mM NADPH, 20 mM GSSG, and 80 *μ*L of enzyme extract. The change in the absorbance was monitored at 340 nm using a UV-visible spectrophotometer (UV-160A, Shimadzu, Kyoto, Japan). One unit of GR was defined as the amount of enzyme needed to oxidise 1 *μ*mol of NADPH in 1 min. The specific activity was expressed as units per gram of FW.

### 2.6. Statistical Analysis

All measurements were performed on 6 experimental replicates, and the results were reported as the means ± the standard errors. A statistical analysis was performed using the Statistical Analysis System program version 6.12 (SAS Institute Inc., Cary, NC, USA). The data were analysed using analysis of variance (one-way ANOVA). The sources of variation were the types of treatments and the storage duration. The means were compared using the least significant differences (LSD) test at a significance level of 0.05. 

## 3. Results and Discussion 

### 3.1. Total Phenolic Content

Phenolic compounds comprise the largest category of phytochemicals in plants, which include flavonoids, phenolic acids, and phenols. These compounds are excellent antioxidants due to their structure, which allows them to easily donate a hydrogen atom to free radicals, and they are the primary molecules responsible for the antioxidant capacity of fruit [24]. Humans cannot produce phenolic compounds; therefore, the main source of these compounds is the consumption of vegetables and fruits [25]. Peppers are an excellent source of phenolic compounds because they have the highest antioxidant capacity among all of the commonly consumed vegetables [26].

**Figure 1:**
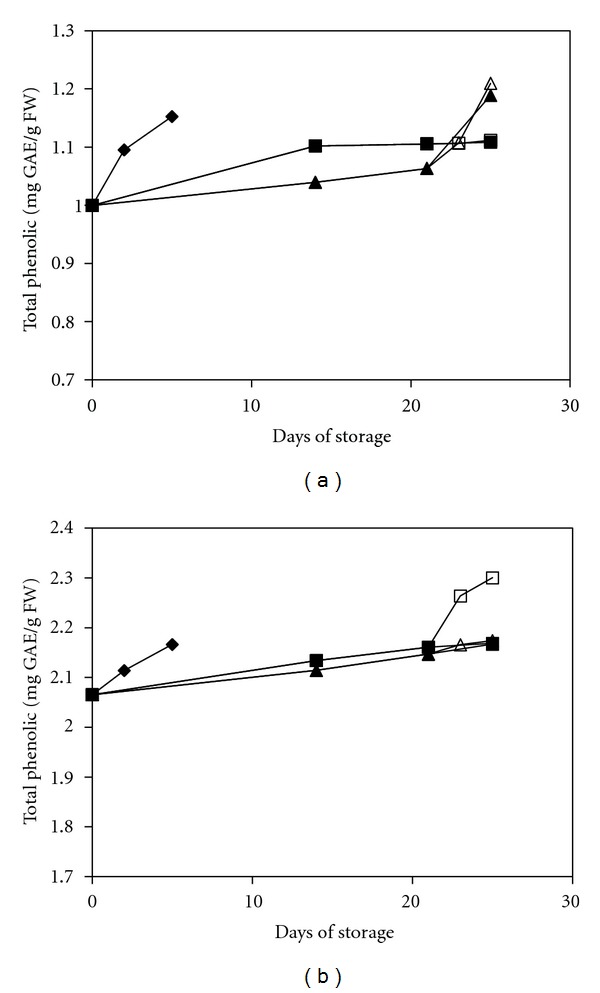
Changes in the total phenolic content of mature green peppers (a) and fully ripe/red peppers (b) for the UCRT (♦), MAP (■), and MAP+1-MCP (▲) treatments during storage at 10°C; MAP (□) and MAP+1-MCP (∆) values for the fruits transferred to room temperature at day 21. The values are the means of six replicate samples, and their S.E.s are indicated.

In the current study, mature green and red peppers showed a gradual increase in the level of total phenolic compounds during low-temperature storage (Figure 1). The phenolic content of the MAP- and MAP+1-MCP-treated peppers was lower than that of the UCRT group at an early stage of storage but higher than that of the UCRT group after 3 weeks of storage. The phenolic content in both types of treated groups was approximately 0.15 mg/g FW higher than in the UCRT group at the end of the storage period. Generally, treatment with MAP or MAP+1-MCP resulted in a delayed accumulation of phenolic compounds during the low-temperature storage. These results indicated that treatment with MAP or MAP+1-MCP successfully increased the tolerance of the fruit to low temperatures and consequently led to a lower activity of chilling-induced phenylalanine ammonia lyase (PAL), the main enzyme responsible for the biosynthesis of phenolic compounds. This conclusion was supported by the finding of Lafuente et al. [27], who stated that PAL is a cold-responsive enzyme; appropriate conditioning that induced cold tolerance would lead to a suppressive effect on PAL activity. According to Faragher and Chalmers [28], the biosynthesis of phenolic compounds is also closely correlated with ethylene production, although the details of this correlation remain unknown. Therefore, the inhibitory effect of 1-MCP treatment on ethylene action might be the reason for the lower level of phenolic compounds in the MAP+1-MCP-treated fruit compared with the MAP-treated fruit during storage. However, the differences were not significant (*P* < 0.05).

### 3.2. Ascorbic Acid Content

Ascorbic acid is a water-soluble antioxidant that neutralises superoxides, hydroxyl radicals, and H_2_O_2_ [29]. It is a functionally, nutritionally, and biologically active compound in the fruit of a pepper plant [30]. Large variations in ascorbic acid levels have been observed due to differences in the cultivars, harvest stages, postharvest handling, agroclimatic conditions, and analytical methods used [31]. In fact, the stage of harvest, storage conditions and duration of storage are the major factors that determine the ascorbic acid concentration in harvested peppers. 

**Figure 2:**
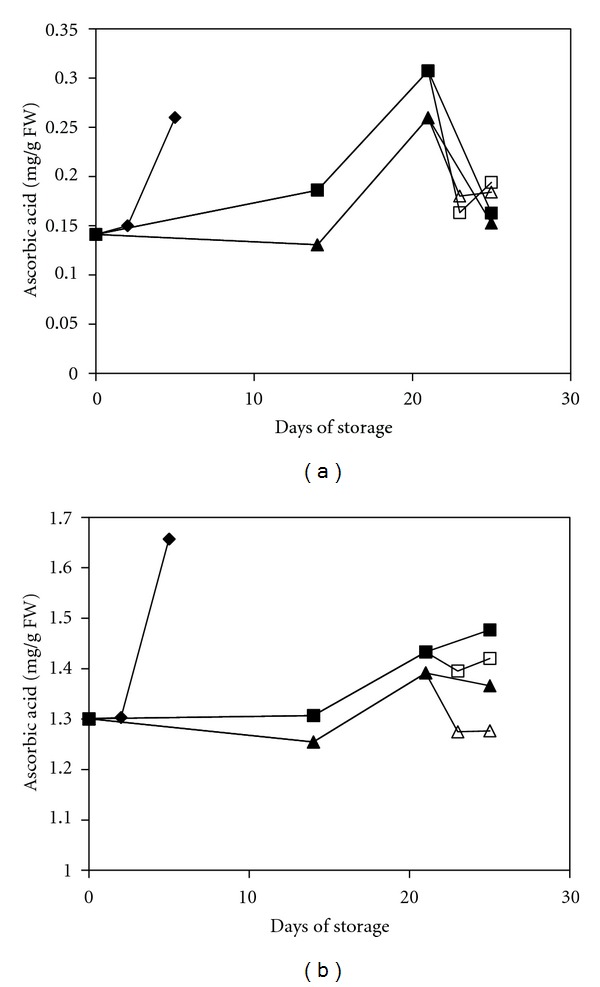
Changes in the ascorbic acid content of mature green peppers (a) and fully ripe/red peppers (b) for the UCRT (♦), MAP (■), and MAP+1-MCP (▲) treatments during storage at 10°C; MAP (□) and MAP+1-MCP (∆) values for the fruits transferred to room temperature at day 21. The values are the means of six replicate samples, and their S.E.s are shown.

The ascorbic acid content was generally higher after the harvest. However, the accumulation of ascorbic acid was delayed in the MAP- and MAP+1-MCP-treated fruits that were stored at low temperature compared with the UCRT group. The elevation of the ascorbic acid content in green peppers did not persist, and the ascorbic acid content decreased rapidly after 21 days (Figure 2(a)). Conversely, the ascorbic acid content in red peppers was consistently low but exhibited a constant elevation throughout the storage period (Figure 2(b)). Currently, little information is available regarding changes in ascorbic acid concentrations during storage and the mechanisms controlling ascorbic acid production. However, light intensity and temperature are known to be the most important factors in determining the final ascorbic acid content [32]. The accumulation of ascorbic acid during the first 3 weeks of storage suggested that high ascorbic acid levels might be a self-protective response against chilling stress, and the delay in the accumulation rate of ascorbic acid proved that the development of chilling stress was successfully suppressed as a result of the treatment. Separately, the storage duration might be the cause of the rapid decrease in ascorbic acid content at the later stages of storage [33]. Nevertheless, Win et al. [34] reported that a high concentration of 1-MCP might have a suppressive effect on the ascorbic acid content in fruit, which suggested a possible reason for the significant difference (*P* < 0.05) observed between the MAP-treated and the MAP+1-MCP-treated green peppers. Furthermore, the difference between the treatment groups might also be due to the additional protection effect of the 1-MCP treatment against chilling stress. Although the fruits were protected from the effect of oxidative stress, the nutritional values were also depleted at the same time due high consuming rate of ascorbic acid in scavenging activities. 

### 3.3. Total Glutathione Content

Reduced glutathione, GSH, is a tripeptide molecule that exists interchangeably with oxidised glutathione, GSSG. GSH plays a key role in many biological mechanisms, including amino acid transport, the biosynthesis of DNA and proteins, and, most importantly, the protection of cells from oxidation. Glutathione is directly involved in the APX-GR system to remove reactive oxygen species (ROS) and maintains a highly reduced intracellular environment [35]. 

**Figure 3:**
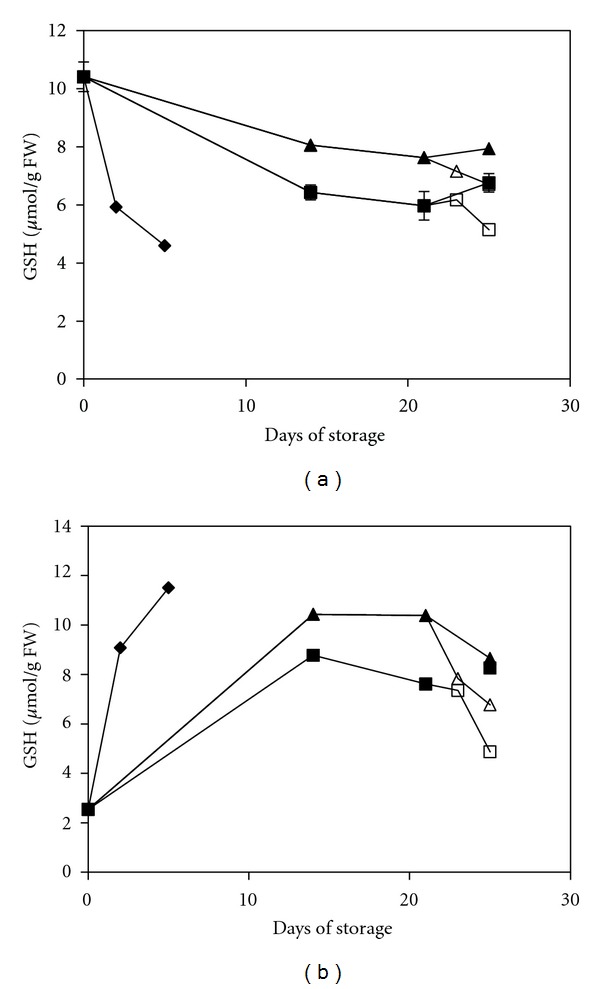
Changes in the GSH content of mature green peppers (a) and fully ripe/red peppers (b) for the UCRT (♦), MAP (■), and MAP+1-MCP (▲) treatments during storage at 10°C; MAP (□) and MAP+1-MCP (∆) values for the fruits transferred to room temperature at day 21. The values are the means of six replicate samples, and their S.E.s are shown.

**Figure 4:**
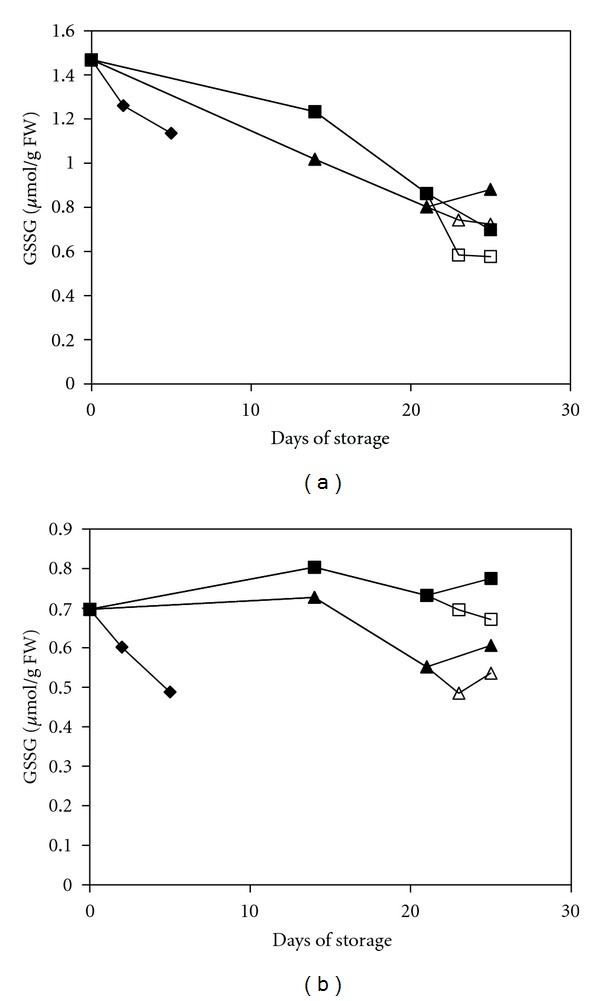
Changes in the GSSG of mature green peppers (a) and fully ripe/red peppers (b) for the UCRT (♦), MAP (■), and MAP+1-MCP (▲) treatments during storage at 10°C; MAP (□) and MAP+1-MCP (∆) values for the fruits transferred to room temperature at day 21. The values are the means of six replicate samples, and their S.E.s are shown.

Our results showed that the GSH content was generally decreased in green peppers but increased in red peppers regardless of the storage temperature or treatment (Figure 3). Clearly, treatment with 1-MCP results in higher levels of GSH in both pepper types compared with the MAP treatment alone, and the difference between these two treated groups was significant (*P* < 0.05) (Table 1). In plants, GSH is the predominant form of glutathione and contains up to 90% of the total glutathione [36]. High ratios of GSH to GSSG are particularly important for a strong defensive mechanism against oxidative stress and to minimise the harm caused by H_2_O_2_ at a cellular level. Conversely, GSSG production was decreased during storage in general (Figure 4). According to an ANOVA analysis, the GSSG content in the MAP+1-MCP-treated group was significantly lower (*P* < 0.05) than that in the MAP-treated group for green peppers. The GSSG content in green pepper was reduced by half by the end of the storage period. However, the difference between the two treated groups in red peppers was not significant. Additionally, the results indicated that the GSH/GSSG ratio in the MAP-treated fruit decreased by 5- to 10-fold; however, in the MAP+1-MCP-treated fruit, the ratio increased by approximately 9- to 18-fold (Table 2). This result implied that the redox status shifted significantly towards a reduced state as a result of the treatment with 1-MCP, which then suppressed oxidative stress and its effect on the cells. A high ratio of GSH/GSSG proved to be important in raising the resistance of fruits against chilling injury [37, 38]. Apart from that, consumption of fruits with high GSH content, such as the MAP+1-MCP-treated pepper, had been proved to be important in enhancing the immune function in human [39].

### 3.4. Enzymatic Antioxidants

The enzymatic antioxidant system, which includes ascorbate peroxidase (APX, EC 1.11.1.11), glutathione reductase (GR, EC 1.6.4.2), and catalase (CAT, EC 1.11.1.6), plays a key role in regulating the defensive response against oxidative stress. The activities of these enzymes in cells were mainly influenced by metabolite specificities, inherent characteristics of the cells, and, most importantly, the environmental factors to which the cells were exposed, such as the level of ROS or the presence of chemicals. Furthermore, these enzymes also exhibit a synergistic interaction with non-enzymatic antioxidants to maintain a reduced environment. The APX-GR system is one of the best examples of this type of interaction. 

**Figure 5:**
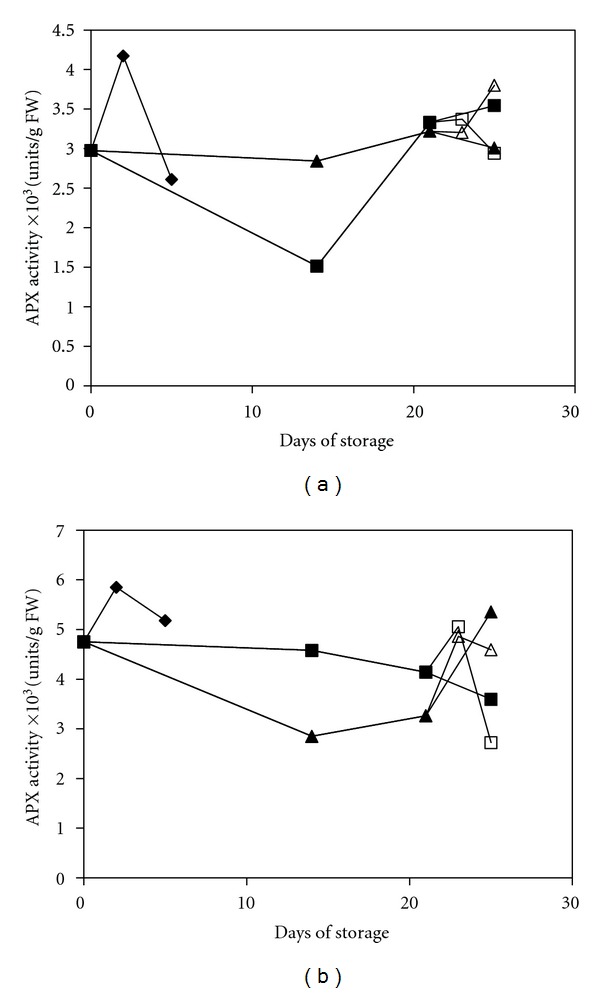
Changes in the APX activity of mature green peppers (a) and fully ripe/red peppers (b) for the UCRT (♦), MAP (■), and MAP+1-MCP (▲) treatments during storage at 10°C; MAP (□) and MAP+1-MCP (∆) values for the fruits transferred to room temperature at day 21. The values are the means of six replicate samples, and their S.E.s are shown.

APX and GR form a closely associated system that effectively removes H_2_O_2_. APX breaks down the H_2_O_2_ that has escaped from the CAT activity or was generated during respiration [40], and GR is responsible for GSH production and ascorbate regeneration [41]. The APX activity was generally lower in the treated fruit compared with that in the UCRT group fruit, especially during the first 2 weeks of cold storage (Figure 5). After two weeks of storage at 10°C, the APX activity in the MAP+1-MCP-treated fruit showed a sustainable increase and eventually reached a higher level than that observed in the UCRT fruit. The resulting APX activity in the MAP+1-MCP-treated fruit was 3.8 kUnits/g FW and 5.8 kUnits/g FW for the mature green peppers and red peppers, respectively, at the end of storage. This finding is in agreement with the study by Singh and Dwivedi [42], which suggested that treatment with 1-MCP provided an additive and sustainable effect to elevate the APX activity. High levels of APX activity might have a suppressive effect on the ascorbic acid levels in the MAP+1-MCP-treated fruit, as was noted earlier in this study. Such observations indicate that the APX activity in the MAP+1-MCP-treated fruit was actively involved in slowing the development of chilling stress during the later stages of storage. Conversely, the APX activity in the MAP-treated red pepper showed a variable change and decreased after two weeks of storage. This result implied that the effect of the MAP treatment alone was not sufficient to sustain the APX activity until the end of storage. 

**Figure 6:**
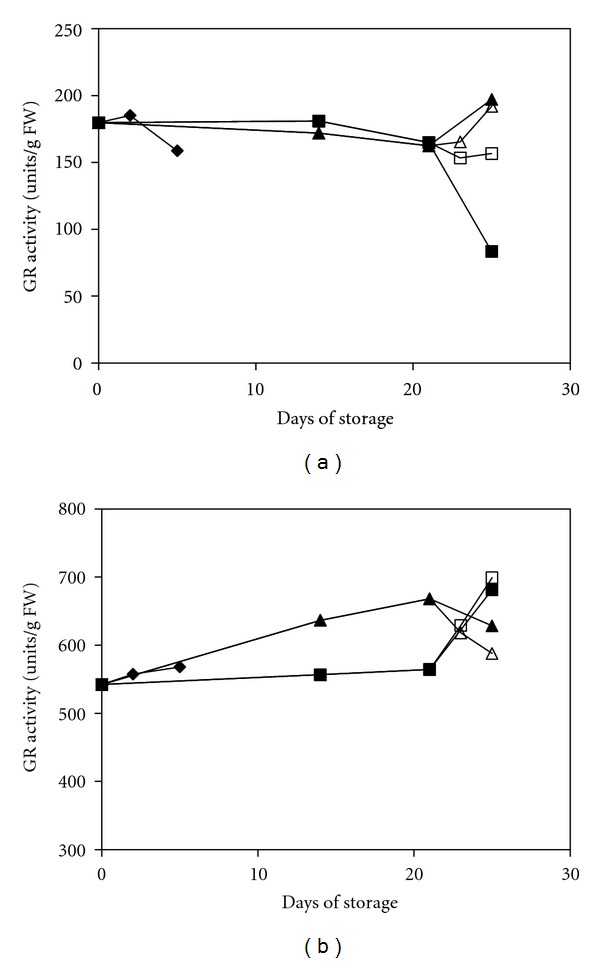
Changes in the GR activity of mature green peppers (a) and fully ripe/red peppers (b) for the UCRT (♦), MAP (■), and MAP+1-MCP (▲) treatments during storage at 10°C; MAP (□) and MAP+1-MCP (∆) values for the fruits transferred to room temperature at day 21. The values are the means of six replicate samples, and their S.E.s are shown.

The GR activity in the treated fruit was likely inconsistent with the change of the GSH content during storage (Figure 6). The GR activity of the MAP- and MAP+1-MCP-treated groups was constantly maintained at a higher level than that of the UCRT group throughout the storage period, especially in the MAP+1-MCP-treated fruit. This higher level was a predictable result because GR is important for sustaining a highly reduced cellular environment by maintaining a high GSH/GSSG ratio [43]. Furthermore, the 1-MCP treatment again showed an additive effect on increasing the GR activity in response to the chilling stress conditions. The difference between the MAP-treated fruit and the MAP+1-MCP-treated fruit was significantly evident (*P* < 0.05) in the red pepper. A similar finding was also observed in cotton plants under stress conditions [44]. Overall, these findings demonstrated that 1-MCP treatment played an important role in regulating the ascorbate-glutathione cycle against oxidative stress. 

**Figure 7:**
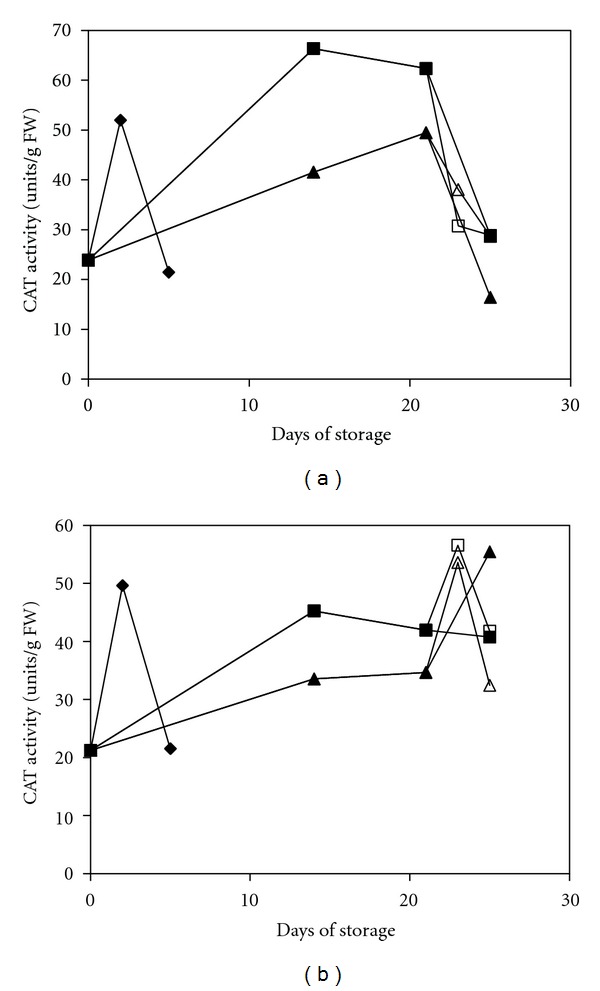
Changes in the CAT activity of mature green peppers (a) and fully ripe/red peppers (b) for the UCRT (♦), MAP (■), and MAP+1-MCP (▲) treatments during storage at 10°C; MAP (□) and MAP+1-MCP (∆) values for the fruits transferred to room temperature at day 21. The values are the means of six replicate samples, and their S.E.s are shown.

CAT catalyses the downstream scavenging system by breaking down H_2_O_2_ [45]. The results obtained in the present study revealed that changes in CAT activity in the treated fruit followed a trend similar to that observed in the UCRT fruit, with both exhibiting an increase in activity at the early stages followed by a decrease (Figure 7). This result is in contrast to the changes in the APX activity, which suggested that the effective removal of harmful substances, such as H_2_O_2_, occurred via a cooperative mediation between APX and CAT. Such modulation is mainly dependent on the levels of substrate (H_2_O_2_) and reductant (ascorbate). CAT has a high catalytic rate but a low affinity towards the substrate, whereas APX has a much higher affinity but requires a sufficient amount of reductant to be activated [21, 46]. Generally, CAT activity in the MAP-treated fruit was higher compared with the MAP+1-MCP-treated fruit. However, in the MAP+1-MCP-treated red pepper, the CAT activity was consistently increased throughout the storage period, which indicated that the 1-MCP treatment might be potentially effective in retaining the CAT activity. A similar finding was also reported in a study regarding plum fruit [47].

## 4. Conclusion

The postharvest conditions for commonly consumed fruits and vegetables are a topic of concern, especially for highly perishable fruits such as the pepper “Kulai” (*Capsicum annuum* cv. Kulai). The present study showed that treatment with MAP or MAP+1-MCP can effectively delay the chilling injury development at low temperatures and extend the shelf life of a pepper by up to 25 days while retaining the nutritional quality of the pepper. The accumulation of the total phenolic and ascorbic acid contents were delayed as a result of the treatments given. The ascorbic acid content and the CAT activity were lower in the MAP+1-MCP treated fruit. However, treatment with 1-MCP did show an impressive effect by upregulating the APX-GR system, which can contribute to the reduction in oxidative stress caused by storage at low temperatures. The GR activity was consistent with the GSH content in maintaining a high GSH/GSSG ratio, which is important to enhance human immunity. More importantly, the effect of 1-MCP was sustained even after the fruits were transferred to storage at room temperature. The commercial utilisation of this technology is likely to have a dramatic impact on the improvement of the storage and handling of horticultural products at the current stage. With slight modification, this postharvest application of 1-MCP can be beneficial for extending the shelf life and market quality of other vegetables and fruits.

## Figures and Tables

**Table 1:** Effect of MAP and MAP+1-MCP treatment on antioxidant constituents in mature green peppers and fully ripe/red peppers during low-temperature storage.

Green peppers	Treatments			

	MAP+RT^∗^	MAP	MAP+1-MCP+RT	MAP+1-MCP

(1) Content				
Total phenolic	1.09 ± 0.09a	1.08 ± 0.08a	1.08 ± 0.08a	1.07 ± 0.08a
Ascorbic acid	0.19 ± 0.01a	0.20 ± 0.01a	0.16 ± 0.01b	0.16 ± 0.01b
GSH	6.83 ± 0.32c	7.39 ± 0.54bc	7.99 ± 0.25ab	8.51 ± 0.36a
GSSG	0.95 ± 0.08a	1.07 ± 0.09a	0.95 ± 0.08a	1.04 ± 0.09a
(2) Activity				
APX	2.83 ± 0.12a	2.84 ± 0.11a	3.21 ± 0.15a	3.01 ± 0.16a
GR	167.05 ± 12.02b	152.19 ± 13.04b	174.19 ± 12.34a	177.72 ± 15.23a
CAT	42.44 ± 2.63ab	45.34 ± 3.56a	36.32 ± 3.28ab	32.83 ± 2.98b

Red peppers	Treatments			
	MAP+RT	MAP	MAP+1-MCP+RT	MAP+1-MCP+RT

(1) Content				
Total phenolic	2.19 ± 0.28a	2.13 ± 0.15a	2.13 ± 0.15a	2.12 ± 0.17a
Ascorbic acid	1.37 ± 0.08a	1.38 ± 0.09a	1.29 ± 0.07a	1.33 ± 0.06a
GSH	6.23 ± 0.24a	6.33 ± 0.35a	7.03 ± 0.62a	7.30 ± 0.41a
GSSG	0.72 ± 0.06ab	0.75 ± 0.03a	0.59 ± 0.05b	0.65 ± 0.08ab
(2) Activity				
APX	4.25 ± 0.02a	4.27 ± 0.03a	4.08 ± 0.05a	4.08 ± 0.03a
GR	579.31 ± 26.25b	576.16 ± 31.25b	610.56 ± 32.16a	618.83 ± 38.92a
CAT	41.35 ± 2.61a	37.29 ± 3.12a	35.08 ± 2.81a	36.21 ± 3.15a

Values are means ± SE of 5 measurements. Different letters indicate significant differences (LSD test, *P* < 0.05) for the means of any antioxidant constituent across the rows.

^∗^RT represents the fruits that were transferred to room temperature at day 21.

**Table 2:** Change in GSH/GSSG ratio in mature green peppers and fully ripe/red peppers stored at low temperature as affected by the MAP and MAP+1-MCP treatments.

Days	Green peppers	Red peppers

	(GSH)/(GSSG)	(GSH)/(GSSG)

UCRT: 0	7.092	3.654
UCRT: 2	4.699	15.107
UCRT: 5	4.05	23.632
MAP: 14	5.214	10.929
MAP: 21	6.917	10.407
MAP: 25	9.683	10.677
MAP: 23 (RT)^∗^	10.583	10.583
MAP: 25 (RT)^∗^	8.922	7.265
MAP/1-MCP: 14	7.912	14.352
MAP/1-MCP: 21	9.512	18.848
MAP/1-MCP: 25	9.003	14.289
MAP/1-MCP: 23 (RT)^∗^	9.644	16.188
MAP/1-MCP: 25 (RT)^∗^	9.278	12.655

*RT represents the fruits that were transferred to room temperature at day 21.
